# Associations between Gestational Diabetes and Anxiety or Depression: A Systematic Review

**DOI:** 10.1155/2021/9959779

**Published:** 2021-07-27

**Authors:** Hong OuYang, Bo Chen, Al-Mureish Abdulrahman, Ling Li, Na Wu

**Affiliations:** ^1^Department of Endocrinology, Shengjing Hospital of China Medical University, Shenyang, China; ^2^Department of Endocrinology, The First People's Hospital of Kerqin District, Tongliao City, Inner Mongolia, China; ^3^Clinical Skills Practice Teaching Center, Shengjing Hospital of China Medical University, Shenyang, China

## Abstract

Gestational diabetes mellitus (GDM) pregnant women are under more psychological stress than normal pregnant women. With the deepening of the study of gestational diabetes mellitus, research has shown that anxiety and depression are also an important cause of gestational diabetes mellitus. Anxiety and depression can cause imbalances in the hormone levels in the body, which has a serious impact on the pregnancy outcome and blood glucose control of pregnant women with GDM. Therefore, the main purpose of this paper is to provide a systematic review of the association between anxiety, depression, and GDM, as well as the adverse effects on pregnant women with GDM. To this end, we searched the PubMed, CNKI, Embase, Cochrane Library, Wanfang, and Weipu databases. Studies on the incidence of anxiety, depression, and GDM, blood glucose in pregnant women with GDM, delivery mode, and maternal and infant outcomes were included to be analyzed, and the source of anxiety and depression in pregnant women with GDM and related treatment measures were discussed.

## 1. Introduction

Gestational diabetes mellitus (GDM) is defined as carbohydrate intolerance resulting in hyperglycemia with first onset or detection during pregnancy [1], accounting for 86% of hyperglycemia during pregnancy [2]. Compared to healthy pregnant women, pregnant women with GDM are more likely to develop maternal and infant complications and are more likely to develop type 2 diabetes [3], cardiovascular disease, dyslipidemia, and metabolic disorders after delivery [4–6].

The mental health problems of pregnant women, especially the mental state of GDM, a high-risk group, have attracted a great attention from scholars all over the world. Studies in this population show that apart from physiological factors, anxiety and depression are also important causes of gestational diabetes [7]. However, there is no unified conclusion regarding the correlation between anxiety and depression and GDM. On the one hand, the study found that anxiety and depression can lead to chronic hypothalamic-pituitary-adrenal hyperactivity, resulting in increased release of cortisol and insulin resistance [8], increasing risk of developing GDM in pregnant women. At the same time, the diagnosis of GDM may increase the risk of antenatal or postnatal depression through a reverse mechanism [9]. This suggests that there may be a two-way relationship between gestational diabetes and anxiety and depression. However, on the other hand, some studies believe that anxiety and depression do not increase the incidence of GDM in pregnant women [10–13], and the diagnosis of GDM does not increase the risk of prenatal or postnatal depression [14–16]. There is currently no consensus on the relationship between anxiety and depression and GDM. We therefore conducted a systematic review of the relevant literature to further explore the bidirectional relationship between anxiety and depression and GDM. At the same time, we further studied the effects of anxiety and depression on blood glucose and maternal and infant outcomes in pregnant women with GDM and discussed the sources of anxiety and depression and related treatment measures.

## 2. Materials and Methods

Using a computer, we retrieved studies from the PubMed, Chinese Journal Full Text (CNKI), EMBASE, Cochrane Library, Wan Fan, and Wei Pu databases. The retrieval period was from the inception of the databases to March 2021, using a combination of theme words and free words. Search terms included “gestational diabetes mellitus,” “anxiety,” “depression,” “incidence of gestational diabetes mellitus,” “blood glucose,” “delivery mode,” “maternal and infant outcomes,” “influencing factors,” and “treatment measures.” There are no specific restrictions on the type, language, or location of the publication type of each study. Articles not directly relevant to the subject were excluded. The EndNote software was used to handle the proper references.

## 3. Results and Discussion

**Figure 1:**
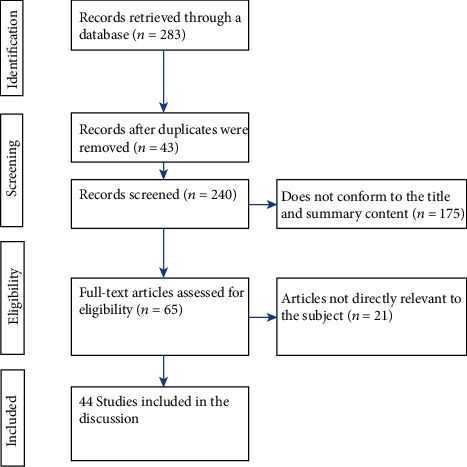
Flow diagram of studies identified.

Following a predesigned literature retrieval strategy, 283 articles were retrieved, including 43 repeated articles. After reviewing the titles and briefs, another 175 were excluded, thus reviewing 65 articles in detail. Finally, we included 44 studies for discussion. The remaining 21 studies were excluded because they were not consistent with our research content. The literature screening process and the results are shown in Figure 1.

### 3.1. Correlation between Anxiety, Depression, and GDM

#### 3.1.1. GDM Is an Associated Factor for Depression or Anxiety

Whether pregnant women with GDM experience higher levels of anxiety or depression than nonpregnant women has not been consistent in some previous studies. On the one hand, research suggests that pregnant women with GDM have been reported to experience higher levels of anxiety and stress than nonpregnant women and healthy pregnant women [17]. Diagnosis of GDM in pregnant women will increase their susceptibility to depression or anxiety [18, 19], resulting in 2-4 times more likely to have antenatal or postnatal depression than women without GDM during pregnancy and after childbirth [20]. According to Egan et al. [21], compared with pregnant women with type 1 diabetes, pregnant women with GDM, and pregnant women without diabetes, it was found that pregnant women with diabetes were more prone to anxiety and depression than pregnant women without diabetes, among whom women with GDM had the highest scores of depression, anxiety, and stress. In a retrospective cohort study [22], Pace et al. found that pregnant women with GDM were twice as likely to be diagnosed with prenatal depression as those without GDM. However, on the other hand, in a large population-based cohort study, GDM was found not to be associated with an increased risk of new-onset mental illness during pregnancy or postpartum [23]. This finding is consistent with the research done by Walmer et al. [24]. For that reason, aiming at existing contradictions, in 2019, Azami et al. conducted the first systematic review and meta-analysis of the relationship between gestational diabetes mellitus and postpartum depression [9]. This meta-analysis included 18 studies, and the final results showed that gestational diabetes significantly increased the risk of postpartum depression [9]. Based on the results of the meta-analysis by Milad, we currently believe that pregnant women with gestational diabetes are more prone to anxiety or depression. However, there is limited research on the relationship between gestational diabetes and postpartum depression, and more research is needed to confirm this.

#### 3.1.2. Relationship between Anxiety and Depression and the Incidence of GDM

In recent years, anxiety and depression among pregnant women are very common [25]. Studies have suggested that anxiety and depression may be a risk factor for the occurrence of GDM [26, 27].

*(1) Anxiety Is Associated with GDM*. Bowers et al. believed that anxiety was a risk factor for the occurrence of GDM [28]; anxiety symptoms were found to significantly increase the risk of developing diabetes [29]. At the same time, Tang et al. conducted a prospective cohort study [30], and the results also showed that the incidence of GDM in pregnant women with anxiety symptoms was higher than that in pregnant women without anxiety symptoms.

*(2) Depression Is Associated with GDM*. The relationship between depression in early pregnancy and GDM is controversial. Tang et al. [30] explored the relationship between depression in early pregnancy (8-14 weeks) and GDM, but did not find that depression in early pregnancy is related to the occurrence of GDM. This is consistent with the results of Katon et al. and Byrn, who believed that the symptoms of depression did not increase the risk of GDM during the first trimester of pregnancy [31, 32]. However, in 2019, a relevant study has found [33] that depressed women are more likely to develop GDM in the first trimester of pregnancy than those who are not depressed. After controlling demographic factors and weight-related variables, depression in the first trimester was still predictive of future GDM development, and depression in the first trimester may indicate an increased risk of subsequent GDM. However, since there is no unified index for the definition of early pregnancy and the diagnosis of depression in various studies at present and the lack of relevant clinical control data, further studies are needed to prove whether depression in early pregnancy will increase the incidence of GDM.

### 3.2. Adverse Outcomes for Any Combination between GDM and Depression or Anxiety

Anxiety, depression, and other negative emotional states can cause imbalances in the body's hormone levels and increase blood sugar, which will have adverse effects on pregnant women with GDM [34, 35]. We summarized the relevant studies (Table 1) to further discuss the effects of anxiety and depression on blood glucose, delivery mode, and maternal and infant outcomes in pregnant women with GDM [21, 33, 36–42].

#### 3.2.1. Influence on Blood Glucose in Pregnant Women with GDM

Studies have suggested that anxiety and depression can cause excitement in the sympathetic adrenal medulla system; promote the secretion of ACTH; increase the levels of glucocorticoid, glucagon, and catecholamine; and finally accelerate the decomposition of gluconeogenesis and glycogen and increase the blood glucose levels of pregnant women [43]. In an earlier randomized controlled study, Wen et al. [39] found that the blood glucose of GDM pregnant women in the anxiety group was higher than that of the nonanxiety group and the blood glucose of GDM pregnant women in the depression group was higher than that of the nondepression group. The results [39] showed that pregnant women with negative emotions tended to have higher blood sugar, which was more difficult to control and those with depression were less optimistic about blood sugar levels. This is consistent with the results of later studies by Horsch et al. [27]. In addition, related studies also found that HbA1c in pregnant women with GDM in the anxiety group was significantly higher than that in the control group [44, 45] and the level of HbA1c was positively correlated with anxiety and depression scores in pregnant women with GDM [27]. It is worth noting that some studies [31, 46, 47] suggest that different levels and types of anxiety were closely related to the HbA1c level. These studies divided GDM pregnant women into a high trait anxiety group and a low trait anxiety group according to the trait anxiety subscale in STAI (used to show the anxiety of personality trait type of patients) and found that the HbA1c of the high trait anxiety group was higher than that of the low trait anxiety group. The mechanism may be that patients with high trait anxiety have a stronger and more persistent response to stressful life events in their daily lives and are more likely to be in a state of high stress [48]. In general, combined with the current relevant studies, we believe that anxiety or depression during pregnancy has adverse effects on blood glucose and HbA1c levels in pregnant women with GDM, and more clinical studies are needed in the future to provide data to further confirm this view.

#### 3.2.2. Influence on Delivery Mode of GDM Pregnant Women

The study found that, compared with the normal group of GDM pregnant women, the anxiety group of GDM pregnant women and the depression group of GDM pregnant women had higher cesarean section and forceps delivery rates, while the vaginal delivery rate was lower [36, 37, 40]. At the same time, the degree of depression in GDM pregnant women was more severe and the rate of vaginal delivery was lower, while the rate of cesarean section and prenatal assisted delivery was higher [40]. The reason why GDM pregnant women in the anxiety and depression group have bad delivery mode may be that anxiety and depression may lead to constant changes in neuroendocrine and intensify the pain of childbirth. At the same time, severe pain response may cause the coordination of uterine contractions to be out of balance, increasing the rate of cesarean section [36, 49]. However, these studies did not mention whether the prepregnancy BMI variable was controlled in pregnant women with GDM. One study [30] believes that there is a positive correlation between BMI and anxiety and depression in pregnant women before pregnancy, suggesting that with the increase of BMI, the incidence of anxiety and depression in pregnant women increases and the incidence of poor delivery methods increases. Therefore, regarding the influence of anxiety and depression on the delivery mode of pregnant women with GDM, we still need more studies to further exclude the role of relevant influencing factors.

#### 3.2.3. Influence on Maternal and Infant Outcomes in Pregnant Women with GDM

Anxiety during pregnancy is a risk factor that leads to adverse outcomes for mothers and infants [10, 50–52]. Pregnant women with GDM experience prenatal depressive symptoms during pregnancy, which may be associated with an increase in adverse pregnancy outcomes [28, 53–55]. As early as 2000, Kurki et al. first reported an association between prenatal depression and an increased incidence of adverse perinatal outcomes in pregnant women [56]. In recent years, a number of studies have found that compared with the normal GDM group, GDM pregnant women in the anxiety and depression group had significantly higher incidences of adverse maternal and infant outcomes, including significantly lower neonatal weight, premature rupture of membranes, postpartum hemorrhage, postpartum infection, macrosomia, neonatal asphyxia, and neonatal hypoglycemia [36, 37, 40]. And there was a positive correlation between the degree of depression and the adverse maternal and infant outcomes in pregnant women with GDM [40]. Such as in 2019, Packer et al. [41] found that women with GDM and depression had significantly higher rates of preeclampsia, gestational hypertension, and preterm birth. The reason may be that depression is associated with vascular changes that may increase the risk of preeclampsia and hypertension [56, 57]. In 2020, Lee et al. [58] found in a cross-sectional study of pregnant women with gestational diabetes in Malaysia that depression, anxiety, or stress in pregnant women with GDM during pregnancy may increase the risk of neonatal morbidity and mortality, eventually leading to adverse neonatal outcomes. In the future, we need to conduct more studies to further explore the relationship between adverse emotions and maternal and infant outcomes.

### 3.3. Related Influencing Factors of Anxiety and Depression in Pregnant Women with GDM

The study on the relevant influencing factors of anxiety and depression in pregnant women with GDM will help us to better understand the source of these negative emotions, provide guidance on intervention measures, improve the prenatal mood of pregnant women, and reduce the impact of anxiety and depression on pregnancy outcomes.

#### 3.3.1. The Influence of Gestational Diabetes Diagnosis on Pregnancy Complicated with Anxiety and Depression

Studies have found that women with GDM are more prone to prenatal depression, anxiety, and stress when they are aware that uncontrolled diabetes can lead to pregnancy-related complications and adverse neonatal outcomes [59, 60]. Once pregnant women know that they have gestational diabetes, one of the sources of anxiety and depression may be that the patient is unable to face their illness correctly and has serious concerns about the disease, which causes certain psychological pressure. Studies have found that the cognition of gestational diabetes among patients is closely related to the occurrence of psychological problems [48, 61]; that is, due to the lack of related professional knowledge of diabetes, the knowledge of diabetes treatment, and the common sense of daily nursing, patients are more worried about the disease, which is more likely to produce anxiety and depression. Another source may come from the need for self-monitoring of blood glucose, dietary restrictions, and the need for insulin treatment [62]. Patients are afraid of treatment and are concerned about the adverse effects of medication on the growth and development of the fetus, resulting in anxiety [15, 48, 63]. Through research [16], Hui et al. divided the sources of anxiety and depression in pregnant women with GDM into three themes: Theme 1—stress related to diagnosis of GDM and perception of high-risk pregnancy; Theme 2—stress associated with losing control of GDM during the dietary management process; and Theme 3—anxiety related to fear of maternal and infant complications. Among them, the study identified concerns about maternal and infant complications (Theme 3) as the biggest source of stress for women with GDM. At the same time, according to this study [16], compared with women who only received dietary treatment, women who received insulin treatment experienced significantly higher levels of stress. This is consistent with the latest study by Lee et al., which found [64] that pregnant women with GDM who received insulin treatment were more likely to have symptoms of anxiety and depression, which may be due to a significant correlation between insulin and hypoglycemia episodes, thus exacerbating patients' concerns about treatment.

#### 3.3.2. The Influence of Various Factors in Pregnant Women with Gestational Diabetes

Women with low family income [65], low socioeconomic status [66], low educational level [66, 67], and high body mass index (BMI) [68] have been reported to have an increased rate of antenatal depression. At the same time, occupation, marital status of pregnant women, and negative life events during pregnancy (daily quarrel) were all related to pregnancy depression. Relevant studies [68, 69] showed that the age of pregnant women with GDM was positively correlated with the prevalence of anxiety and depression, suggesting that the incidence of anxiety and depression increased with age. At the same time, the study found that adverse birth history was positively correlated with anxiety and depression in pregnant women, suggesting that GDM pregnant women with adverse birth history are prone to anxiety and depression [68, 69]. This is consistent with the findings of Tsartsara and Johnson [70]. In addition, Lee et al. [64] found that a family history of depression and anxiety is an important factor in the development of depression and anxiety symptoms, which may be due to the fact that people with a family history of depression and anxiety inherit the genes for psychiatric disorders and may manifest themselves when they are under stress, especially after the diagnosis of GDM. A history of depression was also found to be significantly associated with an increased risk of GDM in a large multiethnic cohort study in the United States [28].

### 3.4. Treatment Measures

In the above studies, we found that pregnant women with gestational diabetes had a higher incidence of anxiety and depression symptoms, suggesting that the mental health level of pregnant women with gestational diabetes was worse than that of normal pregnant women. Anxiety, depression, and other emotions not only affect the outcome of a pregnant woman's own diseases but also harm the growth of the fetus. At the same time, the aggravation of the disease and complications will also lead to or aggravate these bad emotions, forming a vicious circle. Therefore, in addition to the application of drug therapy, pregnant women with GDM also need medical staff to pay attention to the negative emotions of pregnant women with GDM, to carry out targeted psychological counseling for pregnant women, and to take more effective intervention measures as soon as possible to avoid the occurrence of adverse pregnancy outcomes and promote maternal and child health. Relevant studies have shown that comprehensive psychological intervention can help patients to understand gestational diabetes correctly, enhance patients' confidence, and actively cooperate with the treatment of medical staff [15, 48]. Individualized health education can help patients understand the relevant professional knowledge of diabetes, so that patients can see gestational diabetes correctly and thus improve their confidence in treatment [71, 72]. Supportive measures, including spiritual comfort and psychological support, can not only create a healthy and pleasant therapeutic atmosphere to help patients reduce psychological stress, improve cognitive level, and eliminate psychosomatic and psychosomatic reactions but can also help medical staff to understand the patient's personality characteristics to develop appropriate psychological and behavioral treatment measures and procedures. At present, for the treatment of pregnant women with GDM, some organizations suggest starting with diet and exercise therapy to try to achieve a normal blood glucose level [73–75]. Therefore, for pregnant women with GDM who have anxiety and depression, a healthy lifestyle is also essential, including a reasonable diet and appropriate exercise. On the one hand, strict diabetic diet control and appropriate exercise can help patients better control blood glucose during pregnancy [76, 77], and good blood glucose control can also enhance patients' enthusiasm for treatment and ease their anxiety. On the other hand, studies have found that diet and exercise can also reduce the occurrence of adverse pregnancy outcomes in pregnant women with GDM [78], so it can also reduce the adverse effects of anxiety and depression to a certain extent.

## 4. Conclusions

In the above study, we found that anxiety or depression during pregnancy increased the incidence of GDM in pregnant women to a certain extent. And the diagnosis of GDM will increase the incidence of anxiety and depression in pregnant women. At the same time, our study also found that compared with normal GDM pregnant women, GDM pregnant women with anxiety and depression were more likely to have adverse outcomes in terms of blood glucose during pregnancy, delivery mode, and maternal and infant outcomes. Further analysis of the source of anxiety and depression in pregnant women with GDM shows that the diagnosis of gestational diabetes and various factors affecting pregnant women with GDM themselves play a role. As a result, with the increase in the incidence of GDM, the psychological problems of GDM patients also deserve attention. Active and effective psychological intervention measures should be taken to help GDM pregnant women pass the pregnancy period safely and achieve better pregnancy outcomes.

## Figures and Tables

**Table 1:** The effects of anxiety and depression on blood glucose, delivery mode, and maternal and infant outcomes in pregnant women with GDM.

Author, year,	Population	Study design	Emotional indicator	The number of each group	Outcomes	Results
Tong, 2016, China [36]	*N* = 180 GDM	RCT	SAS, SDS	Anxiety/depression GDM = 90 No anxiety/depression GDM = 90	Delivery mode; maternal and infant outcomes	Delivery way: anxiety and depression increased the incidence of cesarean section in pregnant women with GDM Maternal and infant outcomes: anxiety and depression increase the incidence of adverse maternal and infant outcomes in pregnant women with GDM, including pregnancy vomiting, perinatal infection, hydramnios, premature rupture of membranes, postpartum hemorrhage, maternal elevated blood glucose, neonatal preterm birth, low weight, hypoglycemia, and neonatal asphyxia
Xu, 2015, China [37]	*N* = 218 GDM	RCT	HAMA, HAMD	Anxiety GDM = 36 No anxiety GDM = 182	Blood glucose delivery mode Maternal and infant outcomes	Blood glucose: in the anxiety group, fasting blood glucose, blood glucose 2 hours after meal, and glycosylated hemoglobin were increased in GDM pregnant women Delivery way: cesarean section rate increased Maternal and infant outcomes: in the anxiety group, adverse maternal and infant outcomes were increased in GDM pregnant women, including postpartum hemorrhage and low neonatal weight
Zhang, 2017, China [38]	*N* = 110 GDM	RCT	HAMA	Anxiety GDM = 18 No anxiety GDM = 92	Blood glucose	Blood glucose: fasting blood glucose, 2 h postprandial blood glucose, and glycosylated hemoglobin of GDM pregnant women in the anxiety group were significantly increased
Wen, 2009, China [39]	*N* = 95 GDM	RCT	SAS, SDS	Anxiety GDM = 50 No anxiety GDM = 45 Depression GDM = 32 No depression GDM = 63	Blood glucose	Blood glucose: fasting blood glucose was increased in the anxiety and depression groups
Gilbert, 2019, Malaysia [33]	*N* = 418 GDM	Cross-sectional study	DASS-21	Depression GDM = 50 Anxiety GDM = 165 Stress GDM = 40	Maternal and infant outcomes	Maternal and infant outcomes: neonatal respiratory distress was positively correlated with depressive symptoms
Zhao, 2018, China [40]	*N* = 78 GDM	RCT	SDS	Depression GDM = 39 No depression GDM = 39	Delivery mode Maternal and infant outcomes	Delivery way: cesarean section rate of GDM pregnant women in the depression group increased significantly Maternal and infant outcomes: adverse pregnancy outcomes were increased in the depression group, including premature rupture of membranes, postpartum hemorrhage, postpartum infection, macrosomia, neonatal asphyxia, and neonatal hypoglycemia
Packer, 2019, America [41]	*N* = 170,572 GDM	Retrospective cohort study		Depression GDM = 2090 No depression GDM = 168482	Maternal and infant outcomes	Maternal and infant outcomes: adverse pregnancy outcomes were increased in the depression group, including preeclampsia, gestational hypertension, and preterm birth
Horsch, 2016, Britain [27]	*N* = 39 GDM, 164 NDP	Cross-sectional study	DASS-21		Delivery mode Maternal and infant outcomes	Maternal and infant outcomes: adverse pregnancy outcomes were increased in the depression group, including preeclampsia, gestational hypertension, and preterm birth
Egan, 2017, Ireland [21]	*N* = 78 GDM, 32 DM-1, 108 NDP	Cohort study	DASS-21		Maternal and infant outcomes	Maternal and infant outcomes: there was no statistically significant association between maternal psychological variables and maternal Hypertension during pregnancy, outcome of birth, preterm delivery, delivery type, or infant Apgar scores

GDM: gestational diabetes mellitus; NDP: nondiabetic pregnancies; DM-1: diabetes mellitus type 1; SAS: Self-Rating Anxiety Scale; SDS: Self-Rating Depression Scale; DASS-21: Depression, Anxiety, and Stress Scale-21; HAMA: Hamilton Anxiety Scale; HAMD: Hamilton Depression Scale; NICU: Neonatal Intensive Care Unit.
